# The prevalence of Internet addiction and its impact on undergraduates’ mental health in Lagos state, Nigeria

**DOI:** 10.4102/jphia.v16i1.1254

**Published:** 2025-10-03

**Authors:** Evbusogie A. Ezekiel, Mobolanle Balogun, Blossom Maduafokwa, Ijeoma Nwohiri, Barine Wika-Kobani, Opeyemi Giwa, Chioma Ibenye-Ugbala, Oluwadamilola Matti, Aisha Abdulkareem

**Affiliations:** 1 Department of Community Health and Primary Care, Lagos University Teaching Hospital, Lagos State, Nigeria

**Keywords:** Internet, Internet addiction, depression, anxiety, stress

## Abstract

**Background:**

Internet addiction (IA) is prevalent among Nigerian undergraduates. This study seeks to explore the broader mental health consequences of IA among diverse groups of students in Nigerian universities.

**Aim:**

This study aims to assess the prevalence of IA among undergraduates in Lagos State and examine its relationship with health conditions such as depression, anxiety and stress.

**Setting:**

The research was conducted in three public tertiary institutions in Lagos State, involving 830 undergraduates aged 18–24 years.

**Methods:**

This cross-sectional study used a structured questionnaire, incorporating the Internet Addiction Test (IAT) and the Depression, Anxiety and Stress Scale (DASS-21). Chi-square tests determined associations and logistic regression identified predictors of IA. Data analyses were performed using IBM^®^ SPSS 25.0.

**Results:**

The prevalence of IA was 73.3%. Certain critical predictors of IA emphasised the role of institutional affiliation, living arrangements, purpose and timing of use and duration of daily internet engagement. There were positive correlations between IA and depression (*r*_s_ = 0.368), anxiety (*r*_s_ = 0.359) and stress (*r*_s_ = 0.401).

**Conclusion:**

The study found a high prevalence of IA among undergraduates, with significant associations with depression, anxiety and stress.

**Contribution:**

The findings underscore the need to raise awareness about IA and also highlight the need for context-sensitive, evidence-based interventions and for universities and policymakers to implement strategies that aim at promoting healthier internet usage, improving mental health services and raising awareness of the risks associated with excessive online activities.

## Introduction

The world is fast becoming a global digitised village, where the Internet plays an integral role in daily activities such as communication, research and entertainment.^1^ While the Internet provides several benefits, excessive use has been linked to a new phenomenon termed ‘Internet addiction’.^1,2^ This term describes the compulsive, uncontrollable use of the Internet, resulting in significant negative consequences on social, academic and personal aspects of life.^1,3^ Internet addiction (IA) is marked by excessive time spent online, obsessive engagement with Internet activities and failed attempts to limit usage. It often leads to anxiety, impaired work performance and strained personal relationships raising concern about IA, especially among adolescents and young adults, who are the most vulnerable population.^4,5^ College and university students exhibit higher rates of IA because of their constant use of the Internet for academic and social purposes. Global research reveals varying prevalence rates of IA, with studies reporting rates as high as 25% among the United States (US) college students, and up to 35.8% among adolescents in non-Western countries.^6^ In Nigeria, the prevalence of IA among university undergraduates ranges from 2.5% to 10%.^7,8,9^

Internet addiction (IA) has significant mental health implications showing strong associations with psychological disorders such as depression, anxiety and stress.^1^ Individuals suffering from IA are often socially isolated and tend to use the Internet as a coping mechanism to escape from feelings of depression or anxiety.^10^ Consequently, the excessive use of the Internet leads to more profound psychological challenges, thus creating a vicious cycle. For instance, studies have demonstrated a positive correlation between depression and IA, with addicted individuals showing higher levels of mood disorders, loneliness and even suicidal ideation.^9,10,11^ Given the rapid expansion of Internet access and its increasing prevalence, particularly in developing countries like Nigeria, understanding the prevalence of IA and its psychological impact is critical to ensuring the well-being of young people, especially university students.^12^ This study contributes socially by addressing a public health concern that affects a significant portion of the population, especially in a rapidly digitising world, where young people are vulnerable to IA and its mental health implications. Evidences from several studies showed that addressing IA can improve mental health and academic performance of undergraduate students.^2,11,13^

The global rise of IA has attracted significant scholarly attention, particularly in Asia and Europe, where links between IA and mental health disorders, such as depression, anxiety and stress, have been well-documented.^2,3,4^ However, research on the psychological effects of IA in Nigeria remains sparse.^14,15^ Existing studies in Nigeria tend to focus primarily on the prevalence of IA in relation to academic performance, with limited exploration of its connection to mental health. Furthermore, most research in Nigeria has been confined to secondary schools or specific departments within tertiary institutions, leaving a gap in understanding the broader mental health consequences of IA among diverse groups of university students.^7,14^ This study aims to fill this knowledge gap by exploring the relationship between IA and mental health disorders such as depression, anxiety and stress among undergraduates in Lagos. By providing a comprehensive view of how IA affects the mental health of students in Nigerian universities, the study will contribute to the growing body of literature on this topic while offering insights that are specifically relevant to the Nigerian context.

## Conceptual framework

**FIGURE 1:**
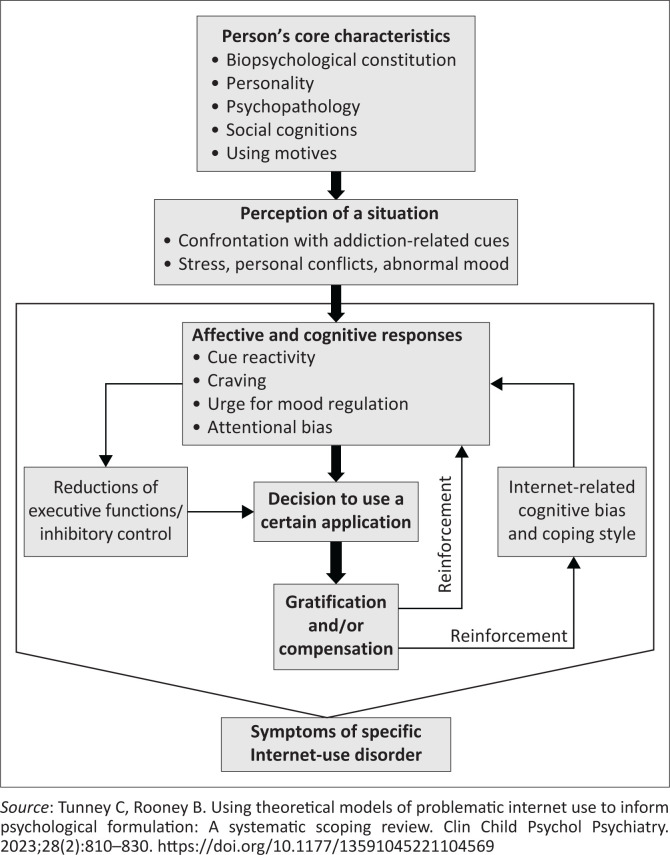
I-Person-Affect-Cognition-Execution Model (I-PACE) adopted from merging theoretical models and therapy approaches in the context of Internet gaming disorder: A personal perspective.

This study is grounded in the theoretical framework of Problematic Internet Use (PIU), which encompasses various forms of compulsive and uncontrolled behaviours associated with excessive Internet use.^16^ According to this framework, IA manifests through behaviours such as compulsive use of social media, online gaming and other online activities that hinder individuals’ ability to manage their time and responsibilities.^16^ The Interaction of Person-Affect-Cognition-Execution (I-PACE) Model offers a comprehensive understanding of how IA develops by examining individual characteristics, emotional states and cognitive control, all of which contribute to IA.^17^ In this context, individuals use the Internet as an escape mechanism from stress or depressive moods, which further reinforces problematic use. This study applied this framework relationship between IA and psychological distress (depression, anxiety and stress) among undergraduates in Lagos, Nigeria (Figure 1).

## Aim and objectives

The aim of this study is to assess the prevalence of IA and its relationship with depression, anxiety and stress among undergraduates in tertiary institutions in Lagos State, Nigeria.

Specific objectives were:

- To assess the prevalence of IA and assess the factors associated with IA among undergraduates in tertiary institutions in Lagos State.
- To examine the relationship between IA and depression, anxiety and stress among undergraduates in tertiary institutions in Lagos State.

## Research methods and design

### Study design

This research utilised a descriptive cross-sectional study design. A cross-sectional study design allowed for the assessment of the prevalence of IA and its relationship with depression, anxiety and stress among undergraduates in tertiary institutions in Lagos State at a single point in time. This design is cost-effective, time-efficient and suitable for identifying relationships between variables in large populations.^1^

### Setting

The study was conducted in public tertiary institutions in Lagos State, Nigeria. Lagos is a densely populated and urbanised economic hub, with a population of approximately 21 million people. It is home to several tertiary institutions, including public universities, polytechnics and colleges for education. The selected institutions for this study were the University of Lagos, Yaba College of Technology and Federal College of Education, Akoka. The setting provided a diverse pool of undergraduates with varying academic and social backgrounds, ensuring a representative sample of the student population in Lagos State.

### Study population and sampling strategy

The study population comprised male and female undergraduates aged 18–24 years, studying full-time at selected public tertiary institutions. The inclusion criteria required that participants had access to the Internet and were in their first to final years of study. Part-time students and students with a prior history of psychiatric illness were excluded. The minimum sample size was calculated using a formula for estimating a single proportion, with a 59.2% prevalence of IA among Nigerian university students (*p* = 0.592).^9^ A non-response rate of 10% was factored into the sample size, leading to a minimum required size of 412 participants. The sample size was doubled to account for design effect, yielding a total sample size of 830 participants.

A multistage sampling technique was employed for the study. At the first stage, one university, one polytechnic and one Federal College of Education were selected through simple random sampling, resulting in the inclusion of the University of Lagos, Yaba College of Technology and the Federal College of Education, Akoka. At the second stage, three faculties were selected from each school by simple random sampling. At the third stage, two departments were selected from each selected faculty by simple random sampling, with the exception of the School of Education at the Federal College of Education, which had only one department. The population of students from each class level in the selected departments were obtained from the deans of the various faculties. Proportionate allocation was used to determine the number of respondents recruited from each institution, and this was further divided proportionately across departments and class levels.

A systematic sampling method was employed to select respondents within each class level. A comprehensive class list (attendance list) served as the sampling frame. The sampling interval (*K*) was calculated using the formula in Equation 1:


K=N/n, [Eqn 1]

where ‘*N*’ is the total number of students in each class and ‘*n*’ is the number of students allocated based on proportionate allocation.

### Data collection

Data were collected using a structured questionnaire, which included the Internet Addiction Test (IAT) to assess the level of IA and the Depression, Anxiety and Stress Scale (DASS-21) to evaluate mental health outcomes. The ‘IAT’ measures the degree of involvement in online activities using responses on a five-degree Likert scale and categorises the addictive behaviour into three categories: mild signs of IA or average Internet use (normal user), moderate signs of addiction and severe addictive behaviour.^10,18^ The total range of the questionnaire was from 20 to 100. A score of 20 to 39 indicated a low level of addiction and average online user; from 40 to 69 represented moderate level of addiction, while a score of 70 to 100 assumed severe level of IA.^19,20^ For this study a score of 40 and above was considered as presence of IA, while a score of 39 and below was considered no IA.^19^ The DASS-21 is an abbreviated version of the DASS-42, developed by Lovibond and Lovibond with a three-dimensional self-reporting scale to assess the presence and intensity of emotional states of depression, anxiety and stress.^18^ The DASS-21 is grounded in a dimensional approach to psychological assessment, as a result, it does not directly correspond to diagnostic classifications like those found in the Diagnostic and Statistical Manual for Mental Health Disorders – 5th Edition (DSM-V) or International Classification of Diseases – 10th Edition (ICD-10).^19^ The IAT demonstrated strong reliability and internal consistency, with Cronbach’s alpha values ranging from 0.90 to 0.95 across various countries and populations.^20^ Both instruments were adopted for this study and have been previously validated in similar populations.^18,19^

The data collection process involved in-person administration of the questionnaire, with trained field workers facilitating the process. To mitigate potential language barriers, the questionnaire was provided in English, which is widely spoken among university students in Nigeria.

### Data analysis

Data were captured and entered into IBM^®^ SPSS Version 25.0 for analysis. After data cleaning, descriptive statistics were used to determine the prevalence of IA and mental health disorders. Bivariate analysis was conducted to identify associations between IA and sociodemographic variables, using chi-square tests. Logistic regression was applied to identify predictors of IA. Results were presented as odds ratios (OR) with 95% confidence intervals (CI), and a *p* ≤ 0.05 was considered statistically significant. Spearman’s rank-order correlation was used to assess the relationship between IA and depression, anxiety and stress.

### Ethical considerations

Ethical approval was obtained 04 June 2021, from the Health Research and Ethics Committee (HREC) of the Lagos University Teaching Hospital, Idi-Araba, with HREC assigned number: ADM/DCST/HREC/APP/4405. Permission from the various departments and individual consent from each participant were obtained.

## Results

A total of 830 questionnaires were distributed among the study participants, all of which were returned and fully analysed, achieving a 100% response rate.

**FIGURE 2:**
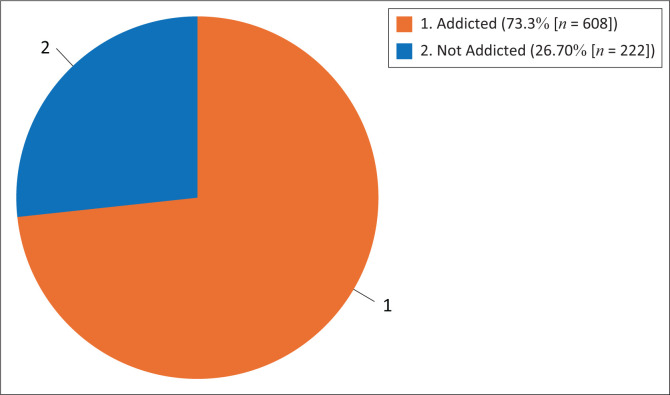
Overall prevalence of Internet addiction among the study participants.

Figure 2 shows the overall prevalence of IA in the study population was 73.3%, which was the cumulative prevalence of moderate and severe classifications of IA.

Table 2 shows that there was a statistically significant association between marital status, religion and living arrangements and IA, *p* = 0.035, *p* = 0.037, *p* = 0.020, respectively. A higher proportion of those who were married or cohabiting were addicted to the Internet (90.0%) than students who were single. A higher proportion of Muslim students (79.5%) were addicted to the Internet than Christian students (71.6%). A higher proportion of students who had other living arrangements (83.3%) were addicted to the Internet than those who lived with their parents or guardians (76.9%) and the majority of students who lived with their parents or guardians (76.9%) were more addicted to the Internet than those who lived on-campus (67.9%) or off-campus (66.9%). There was no statistically significant association between age and sex of the students and IA. Likewise, there was no statistically significant association between pocket money and IA.

**TABLE 1:** Factors associated with Internet addiction among the study participants (socio-demographic characteristics).

Socio-demographic characteristics	Internet addiction				*χ* ^2^	*p*-value
	Addicted (*N* = 608)		Not addicted (*N* = 222)			
	*n*	%	*n*	%		
**Age (years)**						
≤ 20	361	74.7	122	25.3	1.306	0.253
> 20	247	71.2	100	28.8	-	-
**Sex**						
Male	264	75.2	87	24.8	1.193	0.275
Female	344	71.8	135	28.2	-	-
**Marital status**						
Single	581	72.6	219	27.4	4.455	0.035*
Married or cohabiting	27	90.0	3	10.0	-	-
**Ethnicity**						
Yoruba people	441	75.3	145	24.7	5.937	0.115
Igbo people	105	66.0	54	34.0	-	-
Hausa people	9	81.8	2	18.2	-	-
Others	53	71.6	21	28.4	-	-
**Religion**						
Christianity	472	71.6	187	28.4	4.334	0.037*
Islam	136	79.5	35	20.5	-	-
**Estimated monthly stipend (Naira)**						
≤ 10 000.00	198	73.9	70	26.1	7.057	0.133
11 000.00 – 20 000.00	214	69.3	95	30.7	-	-
21 000.00 – 30 000.00	125	75.3	41	24.7	-	-
> 30 000.00	38	86.4	6	13.6	-	-
**Current living arrangement**						
With parents or guardian	369	76.9	111	23.1	9.860	0.020*
On-campus	125	67.9	59	32.1	-	-
Off-campus	99	66.9	49	33.1	-	-
Others	15	83.3	3	16.7	-	-

* , Statistically significant *p* < 0.05.

**TABLE 2:** Prediction of Internet addiction among the study participants.

Predictors of Internet addiction	AOR	95% CI AOR		*p*-value
		Lower limit	Upper limit	
**Marital status**				
Single	0.284	0.080	1.008	0.051
Married or cohabiting	1 (Reference)	-	-	-
**Religion**				
Christianity	1 (Reference)	-	-	-
Islam	1.420	0.925	2.179	0.109
**Institution**				
University of Lagos	1 (Reference)	-	-	-
Federal College of Education, Akoka	1.588	1.039	2.426	0.033*
Yaba College of Technology	1.946	1.232	3.075	0.004*
**Current living arrangement**				
With parents or guardian	1 (Reference)	-	-	-
On-campus	0.710	0.478	1.053	0.088
Off-campus	0.543	0.352	0.838	0.006*
Others	1.146	0.300	4.374	0.842
**Use of Internet mainly for academics**				
Yes	1 (Reference)	-	-	-
No	1.602	1.100	2.333	0.014*
**Use of Internet for mainly for adult websites**				
Yes	2.150	0.804	5.750	0.127
No	1 (Reference)	-	-	-
**Use of Internet mainly during the morning**				
Yes	1.622	1.105	2.380	0.013*
No	1 (Reference)	-	-	-
**Use of Internet mainly during the night**				
Yes	2.148	1.531	3.013	< 0.001*
No	1 (Reference)	-	-	-
**Hours spent on the Internet**				
< 5 h	1 (Reference)	-	-	-
5 h – 8 h	1.205	0.834	1.742	0.320
≥ 9 h	1.682	1.041	2.716	0.034*

AOR, adjusted odds ratio; CI, confidence interval.

* , Statistically significant *p* < 0.05.

**TABLE 3:** Correlation between depression, anxiety, stress and Internet addiction.

Psychiatric variables	Internet addiction	
	Spearman correlation coefficient	*p*-value
Depression	0.368	< 0.001*
Anxiety	0.359	< 0.001*
Stress	0.401	< 0.001*

* , Statistically significant *p* < 0.05.

The predictors of IA were attending Yaba College of Technology or Federal College of Education, Akoka, living off-campus, not using the Internet for academic activities, using the Internet mainly during the morning and at night and spending more than 9 h on the Internet. Students who attended Federal College of Education, Akoka, were 1.6 times more likely to have IA than students who attended University of Lagos (Adjusted Odds Ratio [AOR] = 1.588, 95% CI = 1.039–2.426, *p* = 0.033). Students who attended Yaba College of Technology were approximately two times more likely to have IA compared to students at the University of Lagos (AOR = 1.946, 95% CI = 1.232–3.075, *p* = 0.004). Students who lived with their parents or guardians were 1.84 times more likely to have IA compared to their off-campus counterparts (AOR = 0.543, 95% CI = 0.352–0.838, *p* = 0.006).

Students who mainly used the Internet for non-academic purposes were 1.6 times more likely to be addicted to the Internet than those who used it mostly for academic purposes (AOR = 1.602, 95% CI = 1.100–2.333, *p* = 0.014). Those who went online mostly during the mornings were 1.6 times more likely to be Internet addicted than those who did not (AOR = 1.622, 95% CI = 1.105–2.380, *p* = 0.013). Students’ use of the Internet mostly at night showed that they were approximately two times more likely to be Internet addicted than those who did not use it more at night (AOR = 2.148, 95% CI = 1.531–3.013, *p* < 0.001). Students who spent more than or equal to 9 h online per day were 1.6 times more likely to have IA compared to those who spent less time online per day (AOR = 1.682, 95% CI = 1.041–2.716, *p* = 0.034).

There was a positive correlation between IA and depression (0.368), IA and anxiety (0.359), and IA and stress (0.401), and these were all statistically significant (*p* < 0.001).

There is a moderate positive correlation between IA and depression (0.368). This means that as levels of IA increase, symptoms of depression also tend to increase.

The moderate positive correlation between IA and anxiety (0.359) indicates that individuals who report higher levels of IA are also more likely to experience symptoms of anxiety.

The strongest correlation was with stress (0.401), which also showed moderate correlation to IA.

## Discussion

This study aims to assess the prevalence of IA and its relationship with mental health conditions such as depression, anxiety and stress among undergraduates in Lagos State. The key findings show that the overall prevalence of IA among the participants was 73.3%. Interestingly, the study also revealed higher levels of IA among married or cohabiting students, Muslim students and students with unstable living arrangements compared to their counterparts. Furthermore, students who used the Internet primarily for non-academic purposes, particularly for accessing adult websites, displayed higher rates of addiction. Additionally, the study found a positive correlation between IA and mental health conditions, including depression, anxiety and stress. Moreover, predictors of addiction included the time of Internet use, with use during morning and night being the significant factors and the duration of daily online engagement, particularly spending more than 9 h online per day.

The prevalence of IA identified in this study is notably higher than that reported in similar studies conducted in other regions. For instance, studies from Ibadan, Lebanon, and in southeast Asia indicated much lower rates of addiction.^21,22,23^ These differences could be attributed to variations in methodologies, demographic factors and the classification criteria used to define IA. In Lagos, the increased access to cheaper data plans, the greater availability of Internet services and the use of Internet for both academic and non-academic purposes likely contribute to the higher addiction rates. This finding aligns with previous research which indicates that tertiary institutions typically account for a large proportion of Internet users because of academic demands and the recreational use of the Internet for social media, entertainment and gaming.^12^

The positive correlation between IA and mental health outcomes such as depression, anxiety and stress, reflects findings from earlier studies,^10,11,24^ reinforcing the idea that excessive Internet use can exacerbate existing psychological conditions. Undergraduates, by virtue of their developmental stage, psychological characteristics and increased independence, are particularly vulnerable to these addictive behaviours.^25^ The link between IA and depression observed in this study is consistent with research showing that individuals with IA often experience mood disorders and exhibit withdrawal symptoms when offline.^18,20,26^ Moreover, anxiety, though weaker in its association with IA, was still positively correlated, suggesting that students may use the Internet as a coping mechanism for stress or anxiety related to academic pressure and social expectations.^11,27^

Another noteworthy finding was the relationship between time spent online and severity of addiction. Students who spent more than 9 h a day online were significantly more likely to be addicted. This is in keeping with studies that demonstrate a direct relationship between the amount of time spent on the Internet and addictive behaviours.^11,28,29^ The frequent use of Internet applications designed to capture prolonged attention – such as social media platforms and adult content websites – further fuels this cycle of excessive use and addiction, reinforcing the psychological gratification provided by these activities.

This study identified several significant predictors of IA among undergraduates in tertiary institutions in Lagos State, Nigeria. The findings reveal that institutional affiliation, residential status, primary purpose and timing of Internet use and daily duration of Internet engagement were independently associated with higher odds of IA. Institutional affiliation emerged as a strong predictor. This may suggest contextual or institutional factors such as campus culture, student support systems, academic workload or Internet accessibility policies that differ between institutions and may influence students’ Internet-using behaviours. This also aligns with other studies that suggest that campus Internet access clearly shapes usage patterns.^30^

Living arrangements also significantly influenced IA. Students who lived off-campus were significantly less likely to be addicted to the Internet than those residing with parents or guardians. This may appear counterintuitive, but could be explained by differences in the Internet access, supervision or autonomy between both the groups. On-campus students or those living with family may have more unrestricted access to the Internet-enabled devices or may use the Internet more for leisure when academic oversight is limited. A Chinese study suggested that off-campus students might face barriers to affordable or stable Internet, potentially lowering addiction rates.^31^

The purpose of Internet use played a critical role. Students who primarily used the Internet for non-academic purposes had 1.6 times higher odds of being addicted than their counterparts who used it mostly for academics. This aligns with previous findings that link excessive non-purposeful browsing, gaming or social media engagement with addictive patterns.^32^

### Strengths and limitations

This study’s strengths lie in its large sample size and the use of validated instruments such as the IAT and the DASS-21, which allowed for reliable assessment of both IA and mental health outcomes. The inclusion of three different tertiary institutions provided a comprehensive view of IA across various educational settings in Lagos State, adding to the robustness of the data.

However, there are also notable limitations. The reliance on self-reported data introduces the potential for response bias, as participants may have under-reported or over-reported their Internet use or mental health symptoms. Additionally, the study’s cross-sectional design means that it captures a single snapshot in time, which limits the ability to establish causality between IA and mental health outcomes. The study also faced logistical challenges because of the ongoing Academic Staff Union of Universities (ASUU) strike at the University of Lagos during data collection, which reduced the number of respondents from that institution, potentially affecting the representativeness of the sample.

### Implications and recommendations

The high prevalence of IA found in this study indicates an emerging public health issue among undergraduates in Nigeria that warrants urgent attention. Universities and policymakers should focus on raising awareness of the risks associated with excessive Internet use, particularly its impact on mental health. Interventions could include educational programmes aimed at promoting healthy Internet use, with an emphasis on responsible usage during high-risk periods such as late at night and early in the morning. Counselling services should be made readily available to students who may be struggling with IA or its associated mental health conditions. Additionally, academic institutions could introduce policies that limit access to addictive Internet applications such as adult websites, particularly during study hours. Residence-based interventions may be useful, encouraging parental engagement and digital supervision for students living at home, while enhancing Internet policy and access control in off-campus accommodations.

Further research is needed to explore the long-term effects of IA on students’ mental health and academic performance as well as the underlying factors that contribute to IA among the university students. A longitudinal study design could be implemented to examine the causal relationship between IA and mental health issues over time. Moreover, policies that address the psychological well-being of students, such as offering workshops on managing stress and anxiety, could help reduce reliance on the Internet as a coping mechanism. Policymakers and educational institutions should also consider the broader socio-economic factors, such as the availability of affordable data plans that may contribute to the high rates of IA and explore ways to mitigate their impact on the students’ well-being.

## Conclusion

This study assessed the prevalence of Internet addiction and its relationship with depression, anxiety and stress among undergraduates in tertiary institutions in Lagos State. The findings revealed a high overall prevalence of IA. These findings underscore the growing public health concern posed by IA, especially among young adults in academic settings. The study also draws attention to critical predictors of IA, emphasising the role of institutional affiliation, living arrangements, purpose and timing of use and the duration of daily Internet engagement. These findings highlight the need for context-sensitive, evidence-based interventions and for universities and policymakers to implement strategies aimed at promoting healthier Internet usage, improving mental health services and raising awareness of the risks associated with excessive online activity. Future research should focus on exploring the causal relationships between IA and mental health outcomes and developing effective interventions to curb the rising rates of addiction among students.
